# 4,5-Dimethyl-2-phenyl-1-(*p*-tol­yl)-1*H*-imidazole

**DOI:** 10.1107/S1600536810040535

**Published:** 2010-10-20

**Authors:** P. Gayathri, A. Thiruvalluvar, N. Srinivasan, J. Jayabharathi, R. J. Butcher

**Affiliations:** a PG Research Department of Physics, Rajah Serfoji Government College (Autonomous), Thanjavur 613 005, Tamilnadu, India; b Department of Chemistry, Annamalai University, Annamalai Nagar 608 002, Tamilnadu, India; c Department of Chemistry, Howard University, 525 College Street NW, Washington, DC 20059, USA

## Abstract

In the title compound, C_18_H_18_N_2_, the imidazole ring is essentially planar [maximum deviation = 0.004 (1) Å] and makes dihedral angles of 68.91 (8) and 20.43 (9)° with the tolyl and phenyl rings, respectively. The dihedral angle between the latter rings is 73.62 (8)°. The crystal packing is stabilized by inter­molecular C—H⋯N hydrogen bonds.

## Related literature

For related structures and applications of imidazole derivatives, see: Gayathri *et al.* (2010*a* ▶,*b* ▶,*c* ▶,*d* ▶).
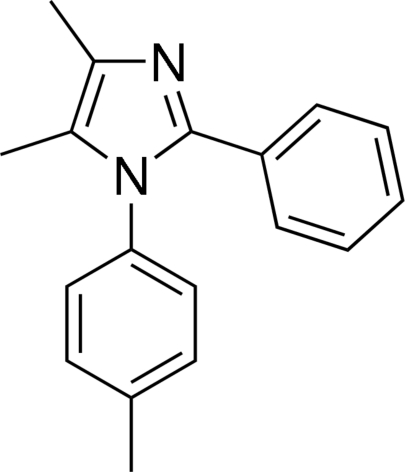


## Experimental

### 

#### Crystal data

C_18_H_18_N_2_
*M* *_r_* = 262.34
Monoclinic, 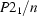

*a* = 9.6971 (3) Å
*b* = 7.5458 (2) Å
*c* = 19.8407 (7) Å
β = 96.604 (3)°
*V* = 1442.16 (8) Å^3^
*Z* = 4
Cu *K*α radiation
μ = 0.55 mm^−1^
*T* = 123 K
0.49 × 0.43 × 0.22 mm

#### Data collection

Oxford Diffraction Xcalibur Ruby Gemini diffractometer
Absorption correction: multi-scan (*CrysAlis PRO*; Oxford Diffraction, 2010 ▶) *T*_min_ = 0.606, *T*_max_ = 1.000
5113 measured reflections
2849 independent reflections
2529 reflections with *I* > 2σ(*I*)
*R*_int_ = 0.024

#### Refinement

*R*[*F* ^2^ > 2σ(*F* ^2^)] = 0.049
*wR*(*F* ^2^) = 0.138
*S* = 1.11
2849 reflections
184 parameters
H-atom parameters constrained
Δρ_max_ = 0.28 e Å^−3^
Δρ_min_ = −0.19 e Å^−3^

### 

Data collection: *CrysAlis PRO* (Oxford Diffraction, 2010 ▶); cell refinement: *CrysAlis PRO*; data reduction: *CrysAlis PRO*; program(s) used to solve structure: *SIR2002* (Burla *et al.*, 2003 ▶); program(s) used to refine structure: *SHELXL97* (Sheldrick, 2008 ▶); molecular graphics: *ORTEP-3* (Farrugia, 1997 ▶); software used to prepare material for publication: *PLATON* (Spek, 2009 ▶).

## Supplementary Material

Crystal structure: contains datablocks global, I. DOI: 10.1107/S1600536810040535/rk2234sup1.cif

Structure factors: contains datablocks I. DOI: 10.1107/S1600536810040535/rk2234Isup2.hkl

Additional supplementary materials:  crystallographic information; 3D view; checkCIF report

## Figures and Tables

**Table 1:** Hydrogen-bond geometry (Å, °)

*D*—H⋯*A*	*D*—H	H⋯*A*	*D*⋯*A*	*D*—H⋯*A*
C12—H12⋯N3^i^	0.95	2.51	3.324 (2)	144
C16—H16⋯N3^ii^	0.95	2.54	3.465 (2)	164

Symmetry codes: (i) 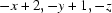; (ii) 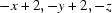.

**Fig. 1.**
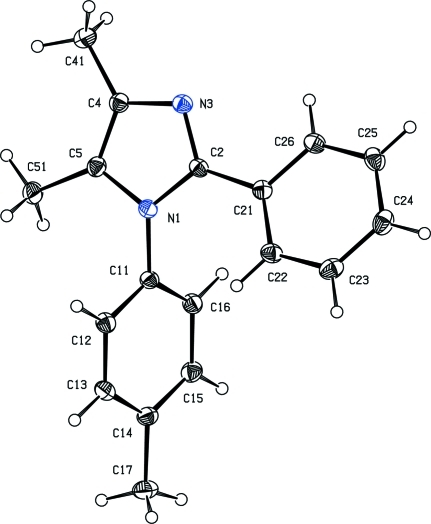
The molecular structure of the title compound, showing the atom-numbering scheme. Displacement ellipsoids are drawn at the 30% probability level. H atoms are shown as small spheres of arbitrary radius.

**Fig. 2.**
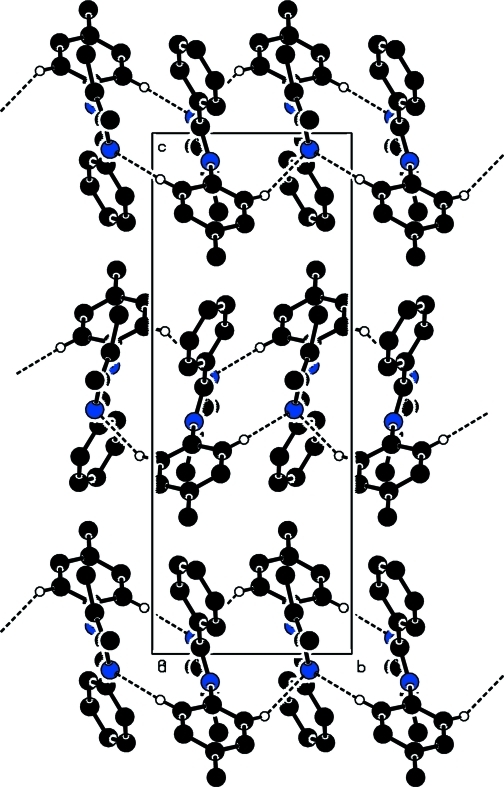
The packing of the title compound, viewed down the a axis. Dashed lines indicate hydrogen bonds. H atoms not involved in hydrogen bonding have been omitted.

## supplementary crystallographic information

### Comment

As part of our research (Gayathri *et al.*, (2010*a*, *b*, *c* & *d*)), we have synthesized the title compound, I and report its crystal structure here.

In I (Fig. 1), C_18_H_18_N_2_, the imidazole ring is essentially planar with maximum deviation = 0.004 (1)Å for N3. The imidazole ring makes dihedral angles of 68.91 (8)° and 20.43 (9)° with the benzene (C11-C16) and phenyl (C21-C26) rings respectively. The dihedral angle between the benzene and phenyl rings is 73.62 (8)°. The crystal packing is stabilized by C12–H12···N3^i^ and C16–H16···N3^ii^ intermolecular non-classical hydrogen bonds (Table 1, Fig. 2). Symmetry codes: (i) -*x*+2, -*y*+1, -*z*; (ii) -*x*+2, -*y*+2, -*z*.

### Experimental

To pure butane-2,3-dione (1.48 g, 15 mmol) in ethanol (10 ml), *p*-toluidine (1.6 g, 15 mmol), ammonium acetate (1.15 g, 15 mmol) and benzaldehyde (1.5 g, 15 mmol) was added about 1 h by maintaining the temperature at 333 K. The reaction mixture was refluxed for 7 days and extracted with dichloromethane. The solid separated was purified by column chromatography using hexane: ethyl acetate as the eluent. Yield: 1.93 g (46%).

### Refinement

H atoms were positioned geometrically and allowed to ride on their parent atoms, with C–H = 0.95 - 0.98Å; *U*_iso_(H) = k*U*_eq_(C), where k = 1.5 for methyl and 1.2 for all other H atoms.

### Crystal data

**Table:**

C_18_H_18_N_2_	*F*(000) = 560
*M**_r_* = 262.34	*D*_x_ = 1.208 Mg m^−^^3^
Monoclinic, *P*2_1_/*n*	Melting point: 388 K
Hall symbol: -P 2yn	Cu *K*α radiation, λ = 1.54184 Å
*a* = 9.6971 (3) Å	Cell parameters from 3666 reflections
*b* = 7.5458 (2) Å	θ = 4.6–74.1°
*c* = 19.8407 (7) Å	µ = 0.55 mm^−^^1^
β = 96.604 (3)°	*T* = 123 K
*V* = 1442.16 (8) Å^3^	Block, colourless
*Z* = 4	0.49 × 0.43 × 0.22 mm

### Data collection

**Table:**

Oxford Diffraction Xcalibur Ruby Gemini diffractometer	2849 independent reflections
Radiation source: Enhance (Cu) X-ray Source	2529 reflections with *I* > 2σ(*I*)
graphite	*R*_int_ = 0.024
Detector resolution: 10.5081 pixels mm^-1^	θ_max_ = 74.2°, θ_min_ = 6.3°
ω scans	*h* = −11→12
Absorption correction: multi-scan (*CrysAlis PRO*; Oxford Diffraction, 2010)	*k* = −8→9
*T*_min_ = 0.606, *T*_max_ = 1.000	*l* = −24→23
5113 measured reflections	

### Refinement

**Table:**

Refinement on *F*^2^	Primary atom site location: structure-invariant direct methods
Least-squares matrix: full	Secondary atom site location: difference Fourier map
*R*[*F*^2^ > 2σ(*F*^2^)] = 0.049	Hydrogen site location: inferred from neighbouring sites
*wR*(*F*^2^) = 0.138	H-atom parameters constrained
*S* = 1.11	*w* = 1/[σ^2^(*F*_o_^2^) + (0.0624*P*)^2^ + 0.7809*P*] where *P* = (*F*_o_^2^ + 2*F*_c_^2^)/3
2849 reflections	(Δ/σ)_max_ = 0.001
184 parameters	Δρ_max_ = 0.28 e Å^−^^3^
0 restraints	Δρ_min_ = −0.18 e Å^−^^3^

### Special details

**Table:**

Geometry. All s.u.'s (except the s.u. in the dihedral angle between two l.s. planes) are estimated using the full covariance matrix. The cell s.u.'s are taken into account individually in the estimation of s.u.'s in distances, angles and torsion angles; correlations between s.u.'s in cell parameters are only used when they are defined by crystal symmetry. An approximate (isotropic) treatment of cell s.u.'s is used for estimating s.u.'s involving l.s. planes.
Refinement. Refinement of *F*^2^ against ALL reflections. The weighted *R*-factor *wR* and goodness of fit *S* are based on *F*^2^, conventional *R*-factors *R* are based on *F*, with *F* set to zero for negative *F*^2^. The threshold expression of *F*^2^ > 2σ(*F*^2^) is used only for calculating *R*-factors(gt) *etc*. and is not relevant to the choice of reflections for refinement. *R*-factors based on *F*^2^ are statistically about twice as large as those based on *F*, and *R*-factors based on ALL data will be even larger.

### Fractional atomic coordinates and isotropic or equivalent isotropic displacement parameters (Å^2^)

**Table:**

	*x*	*y*	*z*	*U*_iso_*/*U*_eq_	
N1	0.96551 (13)	0.70974 (17)	0.05414 (6)	0.0239 (4)	
N3	1.08249 (13)	0.78862 (17)	−0.03075 (7)	0.0248 (4)	
C2	0.95773 (16)	0.7589 (2)	−0.01276 (7)	0.0229 (4)	
C4	1.17482 (16)	0.7599 (2)	0.02618 (8)	0.0260 (5)	
C5	1.10567 (16)	0.7104 (2)	0.07911 (8)	0.0266 (5)	
C11	0.85639 (16)	0.6994 (2)	0.09746 (7)	0.0235 (4)	
C12	0.83053 (17)	0.5406 (2)	0.12883 (8)	0.0272 (5)	
C13	0.73060 (17)	0.5358 (2)	0.17385 (8)	0.0281 (5)	
C14	0.65519 (16)	0.6861 (2)	0.18734 (8)	0.0276 (5)	
C15	0.68211 (17)	0.8429 (2)	0.15400 (8)	0.0302 (5)	
C16	0.78331 (16)	0.8514 (2)	0.10983 (8)	0.0272 (5)	
C17	0.54875 (18)	0.6791 (3)	0.23712 (9)	0.0367 (6)	
C21	0.83051 (16)	0.7676 (2)	−0.06104 (8)	0.0241 (4)	
C22	0.70724 (17)	0.6803 (2)	−0.05163 (8)	0.0289 (5)	
C23	0.59439 (18)	0.6860 (3)	−0.10129 (9)	0.0337 (5)	
C24	0.60157 (19)	0.7807 (3)	−0.16077 (9)	0.0348 (5)	
C25	0.72376 (19)	0.8686 (2)	−0.17049 (8)	0.0334 (5)	
C26	0.83700 (17)	0.8621 (2)	−0.12112 (8)	0.0281 (5)	
C41	1.32767 (17)	0.7884 (3)	0.02550 (9)	0.0341 (5)	
C51	1.15590 (18)	0.6676 (3)	0.15102 (8)	0.0338 (5)	
H12	0.88031	0.43652	0.11971	0.0327*	
H13	0.71349	0.42749	0.19587	0.0338*	
H15	0.63014	0.94623	0.16166	0.0362*	
H16	0.80206	0.95996	0.08843	0.0326*	
H17A	0.48146	0.58494	0.22381	0.0550*	
H17B	0.50045	0.79313	0.23719	0.0550*	
H17C	0.59525	0.65460	0.28267	0.0550*	
H22	0.70063	0.61651	−0.01086	0.0346*	
H23	0.51153	0.62463	−0.09456	0.0404*	
H24	0.52377	0.78531	−0.19451	0.0417*	
H25	0.72962	0.93342	−0.21111	0.0401*	
H26	0.92003	0.92255	−0.12823	0.0338*	
H41A	1.34705	0.91576	0.02429	0.0512*	
H41B	1.35792	0.73153	−0.01470	0.0512*	
H41C	1.37795	0.73656	0.06647	0.0512*	
H51A	1.25562	0.69209	0.15961	0.0507*	
H51B	1.13901	0.54201	0.15960	0.0507*	
H51C	1.10612	0.74057	0.18117	0.0507*	

### Atomic displacement parameters (Å^2^)

**Table:**

	*U*^11^	*U*^22^	*U*^33^	*U*^12^	*U*^13^	*U*^23^
N1	0.0248 (7)	0.0269 (7)	0.0204 (6)	0.0011 (5)	0.0039 (5)	0.0000 (5)
N3	0.0272 (7)	0.0242 (7)	0.0238 (7)	0.0009 (5)	0.0064 (5)	−0.0016 (5)
C2	0.0278 (8)	0.0214 (7)	0.0200 (7)	0.0008 (6)	0.0056 (6)	−0.0013 (6)
C4	0.0262 (8)	0.0248 (8)	0.0273 (8)	0.0020 (6)	0.0047 (6)	−0.0010 (6)
C5	0.0256 (8)	0.0281 (8)	0.0260 (8)	0.0038 (6)	0.0026 (6)	−0.0012 (6)
C11	0.0230 (7)	0.0294 (8)	0.0185 (7)	−0.0001 (6)	0.0036 (6)	−0.0012 (6)
C12	0.0309 (8)	0.0264 (8)	0.0246 (8)	0.0023 (7)	0.0044 (6)	−0.0004 (6)
C13	0.0312 (8)	0.0297 (9)	0.0238 (8)	−0.0032 (7)	0.0045 (6)	0.0025 (6)
C14	0.0231 (8)	0.0374 (9)	0.0224 (8)	−0.0030 (6)	0.0031 (6)	−0.0030 (7)
C15	0.0294 (8)	0.0312 (9)	0.0307 (8)	0.0054 (7)	0.0063 (7)	−0.0025 (7)
C16	0.0295 (8)	0.0261 (8)	0.0263 (8)	0.0014 (6)	0.0043 (6)	0.0014 (6)
C17	0.0295 (9)	0.0477 (11)	0.0347 (9)	−0.0034 (8)	0.0114 (7)	−0.0030 (8)
C21	0.0280 (8)	0.0248 (8)	0.0199 (7)	0.0018 (6)	0.0041 (6)	−0.0043 (6)
C22	0.0314 (8)	0.0328 (9)	0.0233 (8)	−0.0006 (7)	0.0068 (6)	−0.0024 (7)
C23	0.0278 (8)	0.0420 (10)	0.0315 (9)	−0.0018 (7)	0.0045 (7)	−0.0085 (8)
C24	0.0324 (9)	0.0422 (10)	0.0280 (8)	0.0063 (8)	−0.0038 (7)	−0.0077 (7)
C25	0.0417 (10)	0.0367 (10)	0.0216 (8)	0.0039 (8)	0.0026 (7)	0.0000 (7)
C26	0.0321 (8)	0.0296 (8)	0.0234 (8)	−0.0004 (7)	0.0063 (6)	−0.0025 (6)
C41	0.0263 (8)	0.0398 (10)	0.0369 (9)	0.0004 (7)	0.0061 (7)	0.0006 (8)
C51	0.0320 (9)	0.0442 (10)	0.0245 (8)	0.0041 (8)	0.0007 (7)	0.0020 (7)

### Geometric parameters (Å, °)

**Table:**

N1—C2	1.3719 (18)	C24—C25	1.391 (3)
N1—C5	1.392 (2)	C25—C26	1.386 (2)
N1—C11	1.4400 (19)	C12—H12	0.9500
N3—C2	1.319 (2)	C13—H13	0.9500
N3—C4	1.375 (2)	C15—H15	0.9500
C2—C21	1.474 (2)	C16—H16	0.9500
C4—C5	1.362 (2)	C17—H17A	0.9800
C4—C41	1.499 (2)	C17—H17B	0.9800
C5—C51	1.489 (2)	C17—H17C	0.9800
C11—C12	1.386 (2)	C22—H22	0.9500
C11—C16	1.385 (2)	C23—H23	0.9500
C12—C13	1.392 (2)	C24—H24	0.9500
C13—C14	1.392 (2)	C25—H25	0.9500
C14—C15	1.395 (2)	C26—H26	0.9500
C14—C17	1.509 (2)	C41—H41A	0.9800
C15—C16	1.390 (2)	C41—H41B	0.9800
C21—C22	1.396 (2)	C41—H41C	0.9800
C21—C26	1.397 (2)	C51—H51A	0.9800
C22—C23	1.386 (2)	C51—H51B	0.9800
C23—C24	1.388 (3)	C51—H51C	0.9800
			
N3···C12^i^	3.324 (2)	C21···H41A^ii^	3.0800
N1···H22	2.8300	C23···H41B^vi^	3.0400
N3···H26	2.5600	C24···H17A^v^	3.1000
N3···H12^i^	2.5100	C24···H15^vii^	3.0500
N3···H16^ii^	2.5400	C41···H22^i^	3.0800
C4···C22^i^	3.530 (2)	C41···H51A	2.9200
C4···C26^ii^	3.427 (2)	C51···H41C	2.9200
C5···C22^i^	3.537 (2)	C51···H25^iv^	2.8500
C5···C26^ii^	3.362 (2)	H12···N3^i^	2.5100
C11···C22	3.143 (2)	H15···H17B	2.3700
C12···N3^i^	3.324 (2)	H15···C24^vii^	3.0500
C12···C51	3.278 (2)	H16···C2	3.0500
C16···C21	3.530 (2)	H16···N3^ii^	2.5400
C16···C22	3.454 (2)	H17A···H51A^vi^	2.5400
C21···C16	3.530 (2)	H17A···C24^v^	3.1000
C22···C41^i^	3.596 (3)	H17B···H15	2.3700
C22···C4^i^	3.530 (2)	H22···N1	2.8300
C22···C11	3.143 (2)	H22···C11	2.5600
C22···C16	3.454 (2)	H22···C12	2.9700
C22···C5^i^	3.537 (2)	H22···C16	3.0100
C25···C51^iii^	3.537 (2)	H22···C41^i^	3.0800
C26···C4^ii^	3.427 (2)	H23···H41B^vi^	2.4300
C26···C5^ii^	3.362 (2)	H23···C13^v^	2.9300
C26···C51^ii^	3.600 (3)	H25···C51^iii^	2.8500
C41···C22^i^	3.596 (3)	H26···N3	2.5600
C51···C12	3.278 (2)	H26···C5^ii^	2.9600
C51···C25^iv^	3.537 (2)	H41A···C21^ii^	3.0800
C51···C26^ii^	3.600 (3)	H41B···C23^viii^	3.0400
C2···H16	3.0500	H41B···H23^viii^	2.4300
C5···H26^ii^	2.9600	H41C···C51	2.9200
C11···H22	2.5600	H41C···H51A	2.3300
C11···H51C	2.7900	H51A···C17^viii^	3.0700
C12···H51B	2.9800	H51A···C41	2.9200
C12···H22	2.9700	H51A···H17A^viii^	2.5400
C13···H23^v^	2.9300	H51A···H41C	2.3300
C16···H22	3.0100	H51B···C12	2.9800
C17···H51A^vi^	3.0700	H51B···C21^i^	3.0800
C21···H51B^i^	3.0800	H51C···C11	2.7900
			
C2—N1—C5	106.63 (12)	C14—C13—H13	119.00
C2—N1—C11	129.03 (13)	C14—C15—H15	119.00
C5—N1—C11	122.83 (12)	C16—C15—H15	119.00
C2—N3—C4	106.36 (13)	C11—C16—H16	120.00
N1—C2—N3	110.95 (13)	C15—C16—H16	120.00
N1—C2—C21	126.14 (14)	C14—C17—H17A	109.00
N3—C2—C21	122.80 (13)	C14—C17—H17B	109.00
N3—C4—C5	110.17 (14)	C14—C17—H17C	109.00
N3—C4—C41	121.39 (14)	H17A—C17—H17B	109.00
C5—C4—C41	128.41 (15)	H17A—C17—H17C	109.00
N1—C5—C4	105.89 (13)	H17B—C17—H17C	109.00
N1—C5—C51	122.59 (14)	C21—C22—H22	120.00
C4—C5—C51	131.50 (15)	C23—C22—H22	120.00
N1—C11—C12	119.87 (14)	C22—C23—H23	120.00
N1—C11—C16	119.14 (13)	C24—C23—H23	120.00
C12—C11—C16	120.90 (14)	C23—C24—H24	120.00
C11—C12—C13	119.07 (14)	C25—C24—H24	120.00
C12—C13—C14	121.47 (14)	C24—C25—H25	120.00
C13—C14—C15	117.96 (14)	C26—C25—H25	120.00
C13—C14—C17	120.75 (15)	C21—C26—H26	120.00
C15—C14—C17	121.29 (15)	C25—C26—H26	120.00
C14—C15—C16	121.48 (14)	C4—C41—H41A	109.00
C11—C16—C15	119.09 (14)	C4—C41—H41B	109.00
C2—C21—C22	123.89 (14)	C4—C41—H41C	109.00
C2—C21—C26	117.45 (14)	H41A—C41—H41B	109.00
C22—C21—C26	118.58 (14)	H41A—C41—H41C	109.00
C21—C22—C23	120.55 (15)	H41B—C41—H41C	109.00
C22—C23—C24	120.50 (17)	C5—C51—H51A	109.00
C23—C24—C25	119.36 (16)	C5—C51—H51B	109.00
C24—C25—C26	120.24 (15)	C5—C51—H51C	109.00
C21—C26—C25	120.77 (15)	H51A—C51—H51B	109.00
C11—C12—H12	120.00	H51A—C51—H51C	109.00
C13—C12—H12	120.00	H51B—C51—H51C	109.00
C12—C13—H13	119.00		
			
C5—N1—C2—N3	0.36 (17)	N3—C4—C5—C51	−178.79 (17)
C5—N1—C2—C21	176.54 (14)	C41—C4—C5—N1	177.62 (16)
C11—N1—C2—N3	166.40 (14)	C41—C4—C5—C51	−0.7 (3)
C11—N1—C2—C21	−17.4 (2)	N1—C11—C12—C13	176.00 (14)
C2—N1—C5—C4	0.06 (16)	C16—C11—C12—C13	−0.7 (2)
C2—N1—C5—C51	178.60 (16)	N1—C11—C16—C15	−177.22 (14)
C11—N1—C5—C4	−167.05 (13)	C12—C11—C16—C15	−0.5 (2)
C11—N1—C5—C51	11.5 (2)	C11—C12—C13—C14	0.8 (2)
C2—N1—C11—C12	122.48 (17)	C12—C13—C14—C15	0.3 (2)
C2—N1—C11—C16	−60.8 (2)	C12—C13—C14—C17	−179.07 (15)
C5—N1—C11—C12	−73.49 (19)	C13—C14—C15—C16	−1.6 (2)
C5—N1—C11—C16	103.27 (17)	C17—C14—C15—C16	177.82 (15)
C4—N3—C2—N1	−0.62 (17)	C14—C15—C16—C11	1.7 (2)
C4—N3—C2—C21	−176.95 (14)	C2—C21—C22—C23	−176.04 (16)
C2—N3—C4—C5	0.66 (17)	C26—C21—C22—C23	0.7 (2)
C2—N3—C4—C41	−177.56 (15)	C2—C21—C26—C25	176.69 (14)
N1—C2—C21—C22	−19.2 (2)	C22—C21—C26—C25	−0.3 (2)
N1—C2—C21—C26	164.06 (15)	C21—C22—C23—C24	−0.9 (3)
N3—C2—C21—C22	156.57 (15)	C22—C23—C24—C25	0.7 (3)
N3—C2—C21—C26	−20.2 (2)	C23—C24—C25—C26	−0.2 (3)
N3—C4—C5—N1	−0.43 (17)	C24—C25—C26—C21	0.0 (2)

Symmetry codes: (i) −*x*+2, −*y*+1, −*z*; (ii) −*x*+2, −*y*+2, −*z*; (iii) *x*−1/2, −*y*+3/2, *z*−1/2; (iv) *x*+1/2, −*y*+3/2, *z*+1/2; (v) −*x*+1, −*y*+1, −*z*; (vi) *x*−1, *y*, *z*; (vii) −*x*+1, −*y*+2, −*z*; (viii) *x*+1, *y*, *z*.

### Hydrogen-bond geometry (Å, °)

**Table:**

*D*—H···*A*	*D*—H	H···*A*	*D*···*A*	*D*—H···*A*
C12—H12···N3^i^	0.95	2.51	3.324 (2)	144
C16—H16···N3^ii^	0.95	2.54	3.465 (2)	164

Symmetry codes: (i) −*x*+2, −*y*+1, −*z*; (ii) −*x*+2, −*y*+2, −*z*.
